# Decitabine inhibits the cell growth of cholangiocarcinoma in cultured cell lines and mouse xenografts

**DOI:** 10.3892/ol.2014.2499

**Published:** 2014-09-04

**Authors:** BING WANG, HONGBO LI, RUI YANG, SHUNCHANG ZHOU, SHENGQUAN ZOU

**Affiliations:** Department of General Surgery, Tongji Hospital, Tongji Medical College of Huazhong University of Science and Technology, Wuhan, Hubei 430030, P.R. China

**Keywords:** cholangiocarcinoma, decitabine, cell cycle, apoptosis, autophagy

## Abstract

Decitabine (DAC), an inhibitor of DNA methyltransferase, demonstrates antitumor activities in various types of cancer. However, its therapeutic potential for cholangiocarcinoma (CCA), one of the most aggressive gastrointestinal malignancies, remains to be explored. The present study investigated the antiproliferative effects of DAC on CCA cells *in vitro* and *in vivo*. Human CCA cell lines, TFK-1 and QBC939, were used as models to investigate DAC on the cell growth and proliferation of CCA. Cell proliferation was evaluated by Cell Counting Kit-8 assay combined with clonogenic survival assay. Flow cytometry, Hoechst 33342/propidium iodide staining and green fluorescent protein-tagged MAP-LC3 detection were applied to determine cell cycle progression, apoptosis and autophagy. Nude mice with TFK-1 xenografts were evaluated for tumor growth following DAC treatment. DAC was observed to significantly suppress the proliferation of cultured TFK-1 and QBC939 cells, accompanied with enhanced apoptosis, autophagy and cell cycle arrest at G2/M phase. In TFK-1 mouse xenografts, DAC retarded the tumor growth and increased the survival of CCA tumor-bearing mice.

## Introduction

Cholangiocarcinoma (CCA), a highly aggressive malignancy with a growth pattern characterized by periductal extension and infiltration (1), accounts for ~3% of all gastrointestinal malignancies (2). A recent study suggested that the overall incidence and mortality of CCA appears to have increased worldwide over the past decades (3). The prognosis of CCA is poor since patients with CCA are usually at an advanced stage at the time of diagnosis. Complete resection with negative margins is the only treatment with the potential for cure. Although the surgical outcomes and survival rates have gradually improved with the advancement in diagnostic and surgical techniques over the past decades (4), less than one-third of patients present with resectable tumors at diagnosis (5–15). Other treatment options for CCA include adjuvant radiotherapy and chemotherapy and liver transplantation, while none of these approaches have been shown to substantially improve the survival of patients with resected or unresected CCA (16). Thus, novel therapeutic strategies must be developed for the successful treatment of CCA.

Change in DNA methylation represents an important epigenetic alteration during the multistep process of carcinogenesis. DNA hypomethylation leads to genomic instability. Notably, CpG islands in the promoters of tumor suppressor genes are frequently hypermethylated, resulting in inactivation of the corresponding tumor suppressors. Genes that are commonly silenced by promoter hypermethylation are those regulating cell cycle progression, DNA repair, apoptosis and metastasis (17). DNA methylation often occurs at the C5 position of cytosine in a CpG dinucleotide context and is catalyzed by the DNA methyltransferases (DNMTs) (18). DNMT3a and DNMT3b are mainly responsible for *de novo* DNA methylation. DNMT1 maintains DNA methylation by methylating the newly synthesized DNA strand following DNA replication. Unlike genetic mutations, DNA methylation may be reversed by inhibitors of DNMTs (DNMTIs). DNMTIs are therefore emerging as powerful new tools in the epigenetic therapy field. Decitabine (DAC; 5-aza-2′-deoxyazacytidine), one of the well-characterized DNMTIs, functions as a cytosine analog and induces cell death via several mechanisms, including obstruction of DNA synthesis, induction of DNA structural instability and degradation of DNMTs (19). DAC was approved by the USA Food and Drug Administration in 2006 as the standard care for myelodysplastic syndromes (20). DAC may also modulate the response of cancer cells to chemo- and radiotherapy (21). In addition, increasing preclinical and clinical studies have demonstrated a promising application of DAC for the treatment of solid tumors.

To explore the effects of DAC on CCA, the current study used CCA cell lines TFK-1 and QBC939 as models, and investigated the cell proliferation, cell cycle arrest, apoptosis and autophagy following DAC treatment *in vitro*. In addition, an athymic nude mouse model bearing xenografts of TFK-1 cells was examined to test whether DAC inhibits the growth of CCA xenografts.

## Materials and methods

### Cell culture and reagents

The TFK-1 cell line was purchased from DSMZ (Braunschweig, Germany) and the QBC939 cell line was provided by the Third Military Medical University (Chongqing, China). TFK-1 and QBC939 cells were cultured and maintained in a humidified atmosphere containing 5% CO_2_ at 37°C, in RPMI-1640 supplemented with 10% fetal bovine serum and 1% antibiotic-antimycotic (all Gibco-BRL, Carlsbad, CA, USA). DAC was purchased from Sigma-Aldrich (St. Louis, MO, USA).

### Measurement of cell viability

The growth of TFK-1 and QBC939 cells was evaluated by Cell Counting Kit-8 assay (CCK-8; Dojindo, Kunamoto, Japan), according to the manufacturer’s instructions. Briefly, TFK-1 (1×10^4^) and QBC939 (5×10^3^) cells were seeded in 96-well plates. Following incubation with various concentrations of DAC for 24–120 h, CCK-8 solution was added to each well to a final concentration of 10 μl/100 μl medium and incubated for an additional 2 h at 37°C. The absorbance was measured at 450 nm with a reference wavelength of 630 nm by microplate reader (Thermo Multiscan GO, Thermo Fisher Scientific, Waltham, MA, USA).

### Clonogenic survival assay

TFK-1 cells were treated with 0.5, 5.0 or 50.0 μM DAC for five days with culture media changed daily. The cells were then trypsinized, counted and reseeded for clonogenic survival assay on petri dishes at 200 cells per dish. Following incubation at 37°C for three weeks, the cells were fixed with 50% ethanol in ice-cold phosphate-buffered saline (PBS) and stained with 5% crystal violet. The colonies with >50 cells were counted under a microscope (Primo Star, Carl Zeiss, Oberkochen, Germany).

### Cell cycle analysis

Following treatment with various concentrations of DAC for 24, 72 and 120 h, cells were collected and fixed overnight with 70% ethanol (−20°C). At the time of analysis, cells were incubated with 50 mg/ml RNase A for 30 min at 37°C. Following incubation, propidium iodide (PI) was added in the dark to a final concentration of 50 μg/ml. Subsequently, the cell population was analyzed by flow cytometry (BD-LSR; BD Biosciences, Franklin Lakes, NJ, USA).

### Apoptosis detection

Apoptosis was determined by flow cytometry-based assay. Briefly, TFK-1 cells were exposed to DAC at the desired concentration for 24, 48, 72, 96 or 120 h. Apoptosis was evaluated using the Annexin V-FITC apoptosis detection kit (Nanjing KeyGen Biotech., Co., Ltd., Nanjing, China) according to manufacturer’s instructions.

### Hoechst 33342/PI staining

Following incubation with DAC at 25 μM for 120 h, TFK-1 cells were harvested and fixed in methanol for 10 min at room temperature. Following washing with PBS, cells were incubated with Hoechst 33342 (10 μg/ml; Nanjing KeyGen Biotech., Co., Ltd.) and PI (2.5 μg/ml; Nanjing KeyGen Biotech., Co., Ltd.) for 10 min at room temperature. The morphology of the apoptotic cells was observed under a fluorescence microscope (Axio Zoom.V16, Carl Zeiss) and recorded.

### Detection of autophagy with green fluorescent protein (GFP)-tagged MAP-LC3

TFK-1 and QBC939 cells were incubated with DAC for three days and transfected with GFP-tagged MAP-LC3 (GFP-LC3) plasmid. After 24 h, the cells were fixed in 4% paraformaldehyde for 30 min and mounted for confocal microscopy (Leica, Buffalo Grove, IL, USA). GFP fluorescence was observed under a confocal microscope (TCS SP8, Leica). Autophagic cells that showed GFP-LC3 staining were counted.

### Tumor growth and treatment in nude mice

DAC-induced effects *in vivo* with xenografts of TFK-1 cell lines in six-week-old male Balb-c nu/nu mice with a median weight of 14–16 g were evaluated. All animal experiments were performed according to the instructions approved by the Experimental Animal Center of Huazhong University of Science and Technology (Wuhan, China). A total of 10 mice were divided into two groups. All mice were transplanted subcutaneously into the upper right flank with 2×10^6^ TFK-1 cells. Following the detection of a measurable tumor, animals were treated with 0.8 mg/kg DAC or vehicle alone (4% dimethylsulfoxide) by intraperitoneal injection daily for 14 consecutive days. Tumor volumes were calculated every two days using the following formula: Tumor volume (mm^3^)= π/(6xDxd^2^), where ‘D’ is the largest diameter (in mm) and ‘d’ is the smallest diameter (in mm). Mice were monitored daily for treatment-related morbidity and mortality.

### Statistical analysis

Statistical analyses were performed using GraphPad Prism 5 (GraphPad Software, San Diego, CA, USA). All *in vitro* and *in vivo* experiments were repeated independently in triplicate. The Mann-Whitney U test was performed to determine the level of significance for the *in vitro* studies. For *in vivo* studies, the statistical significance was analyzed using the long-rank test. Data are expressed as the mean ± standard deviation, accompanied by the number of tests. P<0.05 was considered to indicate a statistically significant difference.

## Results

### DAC inhibits the growth of CCA cells

**Figure 1:**
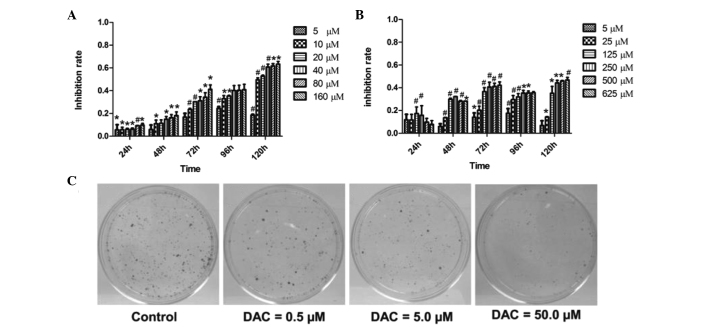
DAC inhibits the cell growth of cholangiocarcinoma cell lines. (A) TFK-1 and (B) QBC939 cells were treated with the indicated concentrations of DAC for the indicated time periods. Relative cell growth inhibition was evaluated by the Cell Counting Kit-8 assay. All assays were performed at least in triplicate. The inhibition rate was in comparison with untreated cells. ^*^P<0.05 and ^#^P<0.01, vs. untreated cells. (C) Clonogenic assay showed the long-term effects of treatment of TFK-1 cells with DAC. TFK-1 cells were treated with the indicated concentrations of DAC for five days. Images of petri dishes from a representative experiment are shown. DAC, decitabine.

To investigate the antiproliferative effects of DAC on CCA cells, the viability of TFK-1 and QBC939 cells treated with various concentrations of DAC was assessed for 24–120 h and the cell viability was determined using CCK-8 assay. DAC was observed to inhibit the proliferation of the two cell lines in a time- and dose-dependent manner (P<0.05; Fig. 1). In TFK-1 cells, treatment with 10 μM DAC for 120 h resulted in 50% suppression of cell proliferation (Fig. 1A). In QBC939 cells, DAC also significantly inhibited the cell growth, but to a lesser extent than in TFK-1 cells (Fig. 1B), indicating that TFK-1 cells are more sensitive to DAC than QBC939 cells. The long-term effect of DAC on CCA cells was assessed by clonogenic assay. Treatment of TFK-1 cells with DAC for five days led to a loss of clonogenicity in a dose-dependent manner (Fig. 1C). As shown in Fig. 1C, the number of tumor clones in the 0.5 μM DAC-treated group was markedly greater than that in the 50.0-μM group. The results demonstrated that DAC may reduce the proliferation of CCA cells.

### DAC induces cell cycle arrest in CCA cells

**Figure 2:**
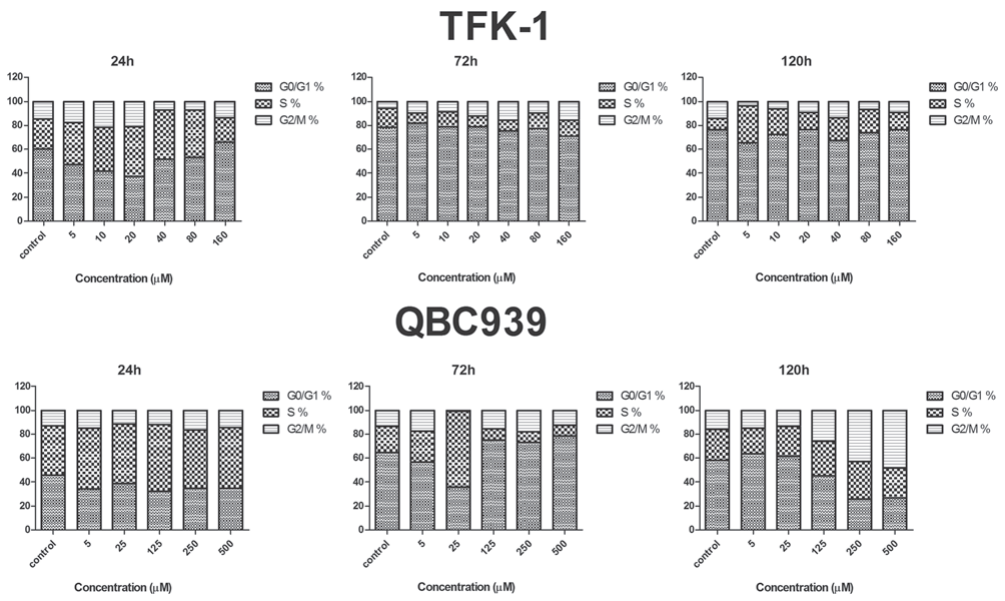
Effects of DAC on cell cycle distribution in cholangiocarcinoma TFK-1 and QBC939 cells. TFK-1 and QBC939 cells were incubated for 24, 72 and 120 h with the indicated concentrations of DAC. Cell cycle distribution was analyzed by DNA content using fluorescence-activated cell sorting. DAC, decitabine.

To determine the mechanism by which DAC inhibits the proliferation of CCA cells, the cell cycle distribution of TFK-1 and QBC939 cells treated with DAC for 24, 72 and 120 h was determined. The percentage of cells in G0/G1, S and G2/M phases are shown in Fig. 2. TFK-1 cells were arrested slightly in G2/M phase in a dose-dependent manner when the DAC concentration was at 40 μM. The cell number in G2/M phase decreased rapidly when the concentration of DAC reached 80 μM, but the cell number in G2/M phase increased slightly when the DAC concentration exceeded 80 μM. Compared with the untreated TFK-1 cells, the accumulation of the cell population in G2/M phase was accompanied by a concomitant decrease in the cell population in G0/G1 phase. By contrast, no apparent alteration of cell cycle distribution was identified in QBC939 cells following DAC treatment for 24 and 72 h. However, following 120 h, an apparent increase in G2/M and decrease in G0/G1 cells was observed following DAC treatment (0–500 μM) in a dosage-dependent manner (Fig. 2; lower panel).

### Inductive effect of DAC on TFK-1 cells apoptosis

**Figure 3:**
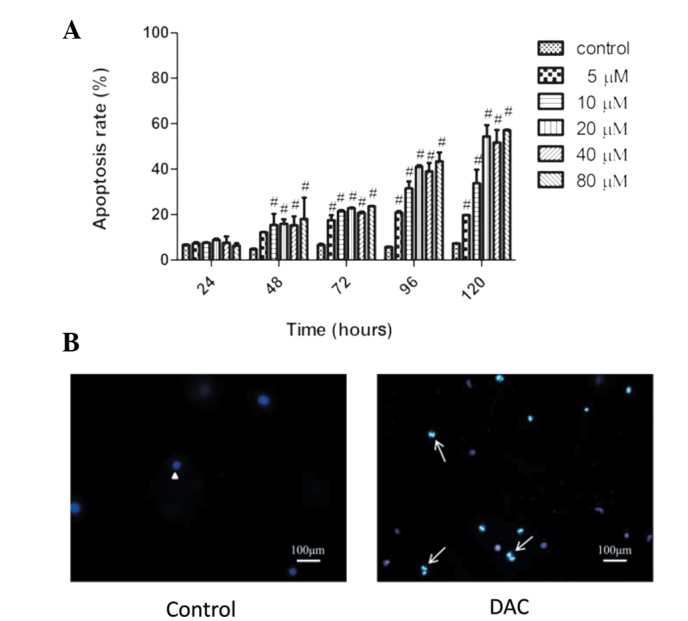
DAC induces apoptosis of TFK-1 cells. (A) Cells were incubated with various concentrations of DAC for the time periods indicated. Apoptosis was measured by Annexin V and PI double-staining. ^#^P<0.01, vs. control. (B) DAC was found to induce morphological changes of apoptosis in TFK-1 cells. Following treatment with 25 μM DAC for 120 h, cells were loaded with Hoechst 33342/PI and then observed using a fluorescence microscope. Normal cells (headed arrow) and apoptotic cells (short arrow) were identified (magnification, ×100). DAC, decitabine; PI, propidium iodide.

The effect of apoptosis in CCA cells following DAC treatment was analyzed. TFK-1 cells were incubated with 0–80 μM DAC for 24–120 h and then stained with Annexin V and PI. DAC significantly induced apoptosis in a time- and dose-dependent manner (Fig. 3A). With the increase of concentration at 120 h, the percentage of apoptotic cells increased from 10.8% in the control group to 57.03% in the 80 μM DAC group. In addition, with the time of incubation, the percentage of apoptotic cells varied from 9.17% at 24 h to 41.59% at 120 h in the 20 μM-treated group. To further support the observed apoptosis by DAC, the apoptotic morphological changes were determined under the fluorescence microscope using Hoechst 33342/PI staining. As shown in Fig. 3B, compared with the control, TFK-1 cells exhibited typical apoptotic features following DAC treatment, including cellular morphological change, apoptotic bodies and condensation of chromatin (indicated by bright blue staining).

### DAC induces autophagy of CCA cells

**Figure 4:**
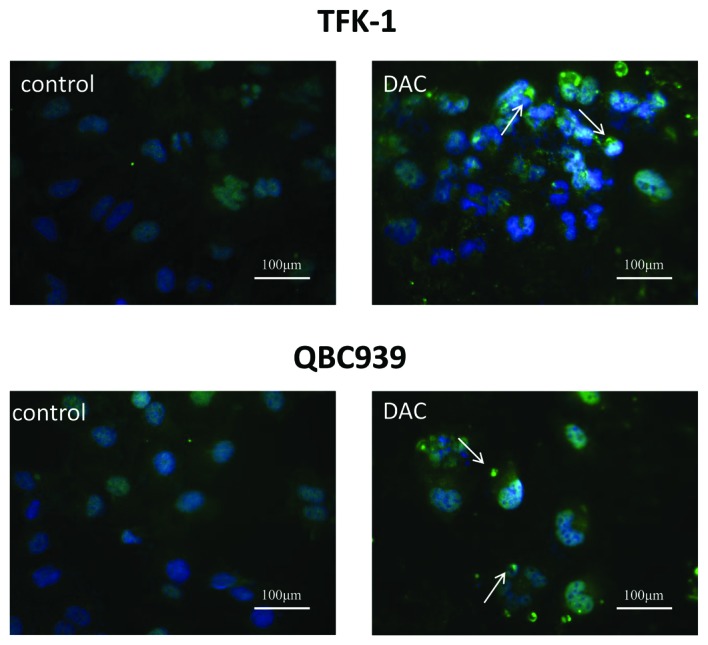
DAC induces autophagy in TFK-1 and QBC939 cells. TFK-1 and QBC939 cells were transfected with GFP-LC3 plasmid and treated with DAC at 25 and 250 μM, respectively. The formation of punctate GFP-LC3 spots was indicative of autophagy (short arrow). DAC, decitabine.

To assess whether a third possible mechanism may contribute to the DAC-induced growth inhibition, autophagic cell death in TFK-1 and QBC939 cells transiently transfected with a GFP-LC3 plasmid was tested. Following treatment with DAC for three days, GFP-LC3 puncta were examined under a confocal fluorescence microscope. In TFK-1 cells, the number of puncta increased from 13 puncta per 100 cells for the control cells to 98 puncta per 100 cells for cells treated with DAC. Similarly, 77 puncta per 100 DAC-treated cells were identified, in comparison with eight puncta per 100 control cells in QBC939 cells (Fig. 4). The results suggested that DAC may induce autophagic cell death in CCA cells.

### DAC reduces the growth of CCA xenografts

**Figure 5:**
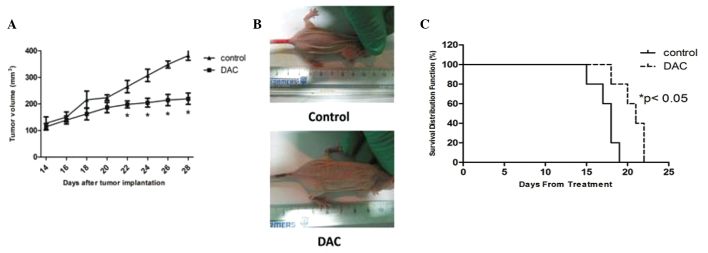
DAC inhibits the growth of cholangiocarcinoma mouse xenografts. (A) Tumor xenografts were established in mice by implanting TFK-1 cells into the upper right flank of the individual mice. Treatment with DAC or control was initiated when the tumors became barely palpable. Animals were treated with 0.8 mg/kg DAC (n=5) or vehicle alone (4% DMSO; n=5) by intraperitoneal injection daily for 14 days. Tumor volume was evaluated every two days until the end of the treatment. Data are presented as the mean ± standard deviation. (B) Representative mice from control and treated groups. (C) Survival was evaluated from the first day of treatment to mortality using the Kaplan-Meier method. ^*^P<0.05, vs. control. DAC, decitabine.

To evaluate the value of DAC therapy *in vivo*, a CCA xenograft mouse model generated by subcutaneous injection of TFK-1 cells into nu/nu mice was used. As shown in Figs. 5A and B, daily administration of DAC (0.8 mg/kg) for a two-week time period was able to reduce tumor growth by ~42.5%. Furthermore, tumor growth inhibition was associated with a significant increase in the survival of DAC-treated animals. The Kaplan-Meier survival curves for each of the two treatment groups are shown in Fig. 5C and the average survival rate of DAC-treated animals was significantly increased. Thus, this clearly demonstrated that DAC exerts a significant antitumor activity against human CCA *in vivo*.

## Discussion

DAC has been widely used as a DNA demethylating agent to reactivate tumor suppressor genes silenced by aberrant promoter hypermethylation. Following phosphorylation by deoxycytidine kinase, DAC is incorporated into DNA. Once in the DNA, DAC is recognized as a target cytosine by the DNMT enzyme. DAC catalyzes the same reaction as normal cytosines with the formation of a covalent intermediate between the catalytic cysteine of the enzyme and 6-position of cytosine analogues (22). DNMT is thereby trapped on the DNA by the suicide inhibitor, triggering DNA repair and degradation of the enzyme. Previously, it has been well documented that DAC exhibits potent antitumor activity, particularly in hematological malignancies. Its therapeutic potential is currently under investigation for treating various types of solid tumor. The majority of previous trials have been designed to determine DAC efficacy on solid tumors in combination with histone deacetylase inhibitors, chemotherapy agents or even stimulators of the immune system (23). In the present study, DAC inhibited the growth of CCA cells in cultured cells and mouse xenografts. CCK-8 assays showed that CCA TFK-1 and QBC939 cells treated with DAC evidently lost cell viability, particularly with the elongated incubation time and increased concentration. However, the TFK-1 cells were more sensitive to DAC than QBC939 cells, suggesting that the inhibitory effect of DAC may be cell type-dependent. Consistently, the colony formation assay showed that DAC could significantly decrease the clonogenic survival of CCA cells, indicating that DAC treatment also produces long-term effects on CCA cell growth.

One of the mechanisms by which antineoplastic agents retard tumor growth is by arresting cell cycle progression. The results of the present study showed that DAC was capable of inducing a G2/M cell cycle arrest in CCA cell lines to a certain extent. Thus, these experimental results indicated that the antitumor effect of DAC on TFK-1 and QBC939 cells is associated with cell cycle arrest.

Additionally, consistent with other solid tumors (23), the results of the current study showed that DAC induces apparent apoptosis in CCA cell lines. TFK-1 cells showed shrinkage and condensation of the nuclear chromatin and cytoplasm. The results of morphological changes were consistent with those of flow percentage of apoptotic cells measured by flow cytometry following Annexin V/PI staining.

Furthermore, Schnekenburger *et al* previously reported that autophagy may be involved in DAC-induced cytotoxicity in human chronic myelogenous leukemias (24). In the present study, compared with the control group, treatment with DAC enhanced the formation of autophagosomes. Therefore, DAC-mediated growth inhibition of CCA cells may also be via induction of autophagy.

In the current study, the effects of DAC on CCA tumor cell lines were also evaluated *in vivo* using a CCA xenograft model. A total of 0.8 mg/kg DAC for two weeks significantly reduced the growth of xenografted TFK-1 cells by 42.5%. In addition, the mice treated with DAC suffered from a comparatively decreased tumor burden and exhibited prolonged survival than that of the control groups. The results are consistent with those from the Lu Z group (25). However, Yi *et al* previously reported that the treatment of endometrial tumors with DAC at a dose of 15 mg intraperitoneally injected thrice weekly for six consecutive weeks was unable to significantly suppress the tumor growth, with the exception of treatment with a combination of DAC and valproic acid (26). The results suggested that different tumor types require different DAC regimens.

In summary, the present study demonstrated that DAC is capable of suppressing the growth of CCA cells *in vitro* and *in vivo*, suggesting a promising therapeutic development of DAC for treating CCA.
